# Carbon Dots as an Effective Fluorescent Sensing Platform for Metal Ion Detection

**DOI:** 10.1186/s11671-019-3088-6

**Published:** 2019-08-13

**Authors:** Donggeon Yoo, Yuri Park, Banyoon Cheon, Myoung-Hwan Park

**Affiliations:** 1 Nanobiomaterials Research Institute, Sahmyook University, Seoul, 01795 Korea; 2 Department of Convergence Science, Sahmyook University, Seoul, 01795 Korea; 3 Department of Chemistry and Life Science, Sahmyook University, Seoul, 01795 Korea

**Keywords:** Carbon dots, Graphene quantum dots, Heavy metal ions, Sensing

## Abstract

Fluorescent carbon dots (CDs) including carbon quantum dots (CQDs) and graphene quantum dots (GQDs) have drawn great interest because of their low cost and low toxicity, and they represent a new class of carbon materials prepared by simple synthetic routes. In particular, the optical properties of CDs can be easily tuned by the surface passivation of the organic layer and functionalization of the CDs. Based on the advantages of these carbon materials, CQDs and GQDs have been applied in various fields as nanoplatforms for sensing, imaging, and delivery. In this review, we discuss several synthetic methods for preparing CQDs and GQDs, as well as their physical properties, and further discuss the progress in CD research with an emphasis on their application in heavy metal sensing.

## Introduction

The discovery of fluorescent carbon dots (CDs), also known as carbon quantum dots (CQDs), has attracted tremendous interest from many researchers because of their versatile applications in optoelectronics, biomedical applications, and chemical biosensors [1–3]. All nano-sized fluorescent carbon materials with one dimension less than 10 nm can be classified as CDs, and these can be derived from various carbon materials such as fullerenes, graphite, carbon nanotubes, and graphene [4–6]. CDs have several advantages compared to other conventional fluorescent sensors. For example, organic dyes are inexpensive and effective as fluorescent probes, but they are easily photobleached. In contrast, CDs are much more resistant to photobleaching [7–9]. Additionally, semiconductor quantum dots (QDs) are comparably as good as CDs in terms of photostability, quantum efficiency, and tunable fluorescence, but QDs cannot be used to trace a single molecule for long-term monitoring because of their intrinsic blinking [10–15]. Moreover, the main pitfall of QDs is their toxicity, which is due to their heavy metal content, including metals such as cadmium; this limits their biological and environmental applications [16–19]. Compared to other fluorescent raw materials, CDs are synthesized from inexpensive carbon sources that are abundant in nature and are, thus, bio-friendly. Furthermore, there are several simple methods to modify the surface state of CDs, which allow researchers to tune the solubility and quantum yields of CDs according to their experimental requirements [20–30].

**Fig. 1:**
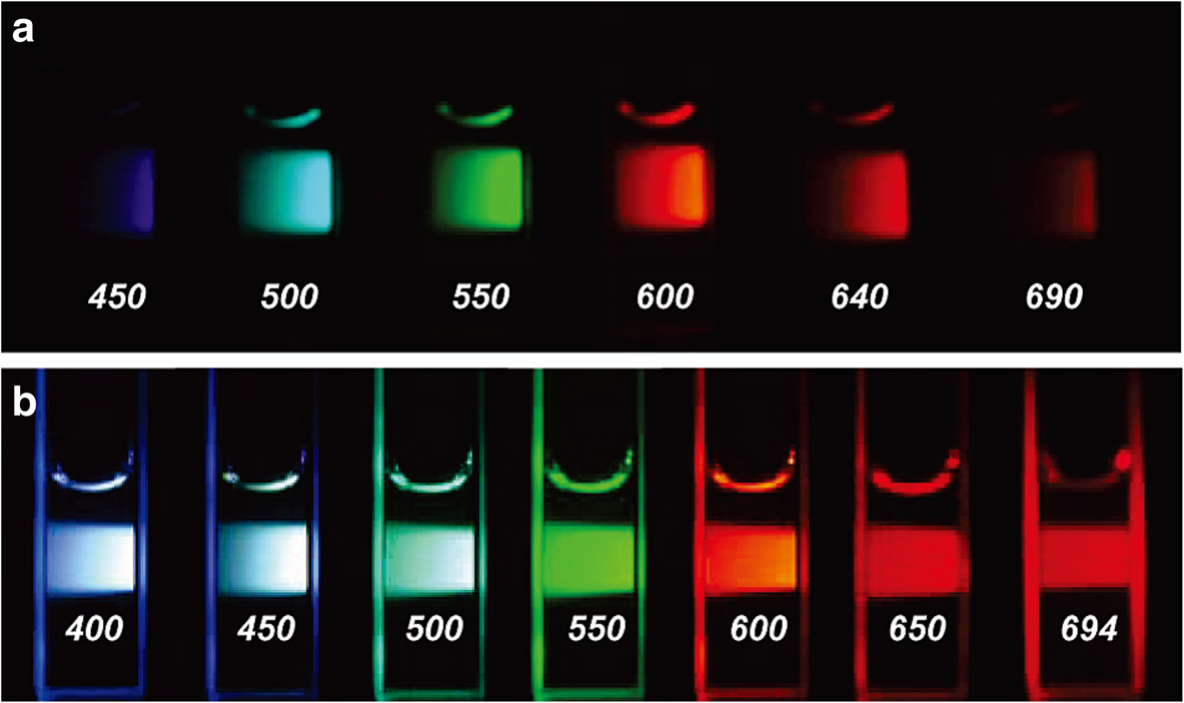
Aqueous solution of diaminopolyethylene glycol (PEG1500N)-attached CDs (**a**) excited at 400 nm and photographed through band-pass filters of different wavelengths (as indicated) and the CDs (**b**) excited at the indicated wavelengths and photographed directly [32]

Among the various possible applications of CDs, here, we summarize how CDs can detect heavy metals, as well as the types of materials that can be utilized. Some heavy metals, such as zinc or iron, are essential for human metabolism and are rarely harmful to human health when in their optimal concentration. In contrast, other metals, such as Hg^2+^, Pb^2+^, and Cd^2+^, are detrimental to humans, even in trace amounts. These toxic metals are easily accumulated in the body and coordinate with biological components, such as enzymes and nucleic acids, hindering normal biological interactions and functions. In this respect, CDs are good candidate components for metal sensors because they are biocompatible. In addition, the fluorescence quantum yield of CDs can be enhanced by modifying the CD surface by adjusting the intrinsic components and surface groups [31]. Herein, we outline the synthetic methods and physical features of CDs reported in early studies, and we summarize the recent progress in using CDs as probes for heavy metals (Fig. 1).

## Synthesis of Carbon Quantum Dots

In the past decades, numerous synthetic methods for the preparation of CDs have been explored. These methods can be largely categorized into two approaches: top-down and bottom-up [33]. Simply, the former process cleaves bulk carbonaceous materials into CDs via physical, chemical, or electrochemical methods, whereas the latter synthesizes CDs from appropriate precursors from various carbon sources. Surface modification can be applied after or during CD synthesis via surface passivation, doping, or functionalization. Because many synthetic procedures have been summarized elsewhere, here, we briefly describe the development and advances in CD research from the early years of their discovery.

### Top-down Methods

**Fig. 2:**
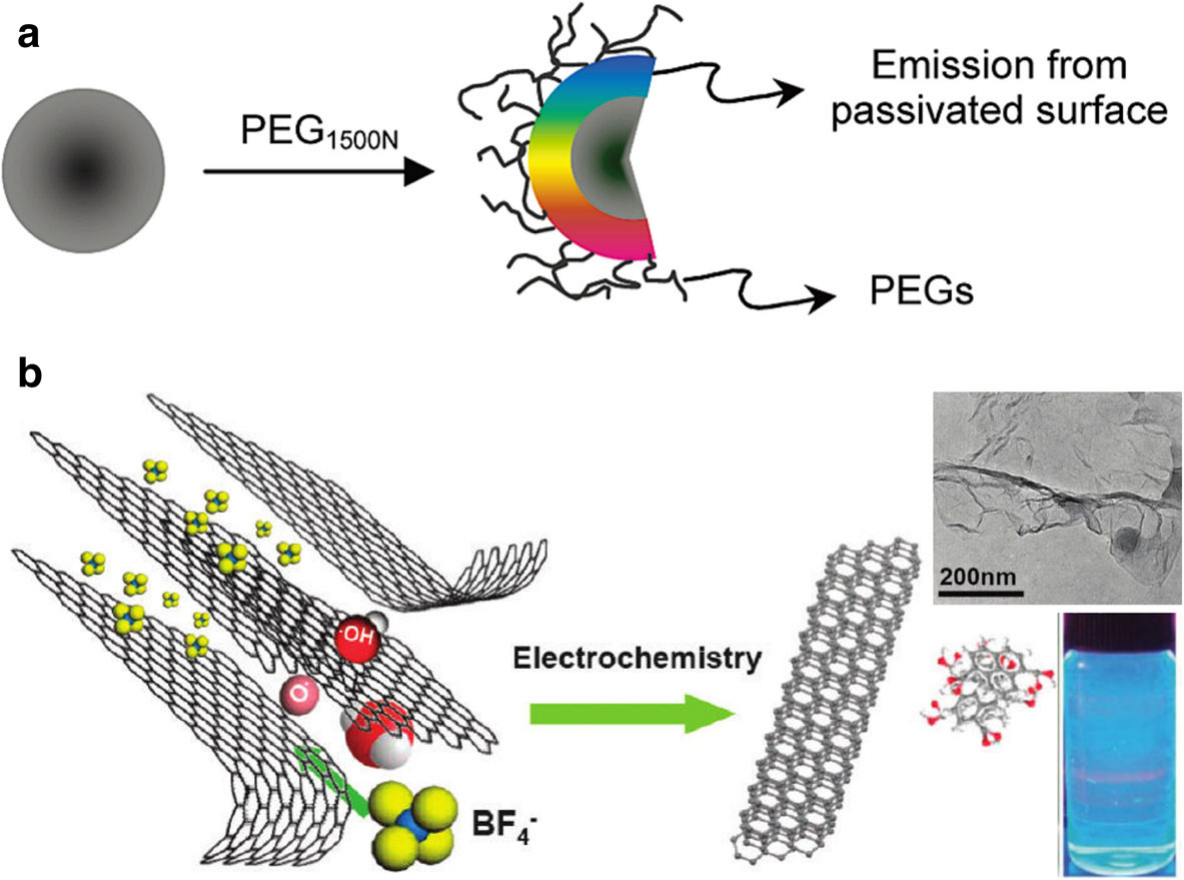
Illustration of the formation of **a** fluorescent CDs via laser ablation with PEG attached to the surface [32] and **b** GCDs prepared by exfoliation in an ionic liquid [36]. The inset is a transmission electron microscopy (TEM) image of the fabricated GCDs and the solution illuminated by an UV lamp

In the top-down method, carbon macromolecules are cut into smaller pieces using physical forces such as arc-discharge, laser ablation, or electrochemical reactions. Subsequently, further surface modification is applied to enhance and tune their fluorescence [34]. CDs were discovered as a byproduct of the synthesis of single-walled nanotubes (SWNTs) prepared by the arc-discharge method [35]. Because of the impurities in the resultant suspension, further electrophoretic separation occurred, and a fluorescent and fast moving band was isolated; these were referred to as fluorescent nanoparticles. Since then, researchers have extended their study to other carbon allotropes, and various modification methods to create a range of fluorescent materials have been reported. Sun et al. reported photoluminescent CDs prepared using laser ablation (Fig. 2a) [32]. These CDs were prepared from the hot-pressing of cement and graphite and cut with laser in a hot vapor-filled chamber. Because the products were composed of various sizes of non-fluorescent particles, further polymeric passivation with diaminopolyethylene glycol (PEG_1500N_) or poly(propionylethyleneimine-*co*-ethyleneimine) (PPEI-EI) was applied to confer the dots with fluorescence. A subsequent study tested whether there was a relationship between the fluorescence of the CDs and the solvent type. Initial CDs were prepared from graphite irradiated with a laser in PEG_200N_/water [37]. Because the CDs prepared from PEG_200N_ were fluorescent, the study concluded that solvents can be used for the functionalization of CDs.

Another study reported another fluorescent CD prepared using multiwall carbon nanotubes (MWCNTs) via an electrochemical method [38]. The MWCNTs were placed between two electrodes in an electrolytic solution, and a voltage was applied at a constant rate. The voltage cycling recurrently led to the oxidization and reduction of the MWCNTs, and this broke down C-C bonds of the MWCNTs, widened defects to allow the incorporation of oxygen, and generated hydroxyl/carboxyl residues. As this reaction progressed, the solution changed from yellow to dark brown and emitted blue light under UV irradiation. The particles were uniformly spherical with a size of 2.8 nm in diameter. Similarly, other CDs have been synthesized from graphite using electrochemical exfoliation, where two graphite electrodes are placed in an alkali electrolyte solution (NaOH/ethanol), followed by the application of a current. The graphite rods are exfoliated into chips and generate fluorescent CDs with a size of 4 nm [39].

Subsequently, researchers tried to develop simpler and more efficient methods of CD synthesis. The selection of electrolytes provides another way to control the properties of the CDs. For example, an imidazole ionic liquid can be used as an electrolyte. This liquid performs two roles, acting as an electron acceptor at the anode and also penetrating the graphite sheet and accelerating the exfoliation process [36]. However, its application generated a range of particle sizes and morphologies, and its removal is complicated and time-consuming.

The generation of fluorescent graphene quantum dots (GQDs) from graphene requires more steps than other types of carbon macromolecules [40]; first, the graphene must be separated from a chunk of graphite by oxidation [41]; subsequently, the graphene oxide (GO) must be cut with various methods as mentioned above [42–44]. Pan’s group reported a simple hydrothermal approach for the cutting of graphene sheet into GQDs with bright blue photoluminescence [45]. In addition, Zhu et al. reported the creation of GQDs with a large-scale zigzag edge structure through acidic exfoliation and etching of pitch carbon fibers [4], and Le et al. prepared fluorescent CDs by the exfoliation of graphite in ionic liquids (Fig. 2b) [46].

### Bottom-up Method

**Fig. 3:**
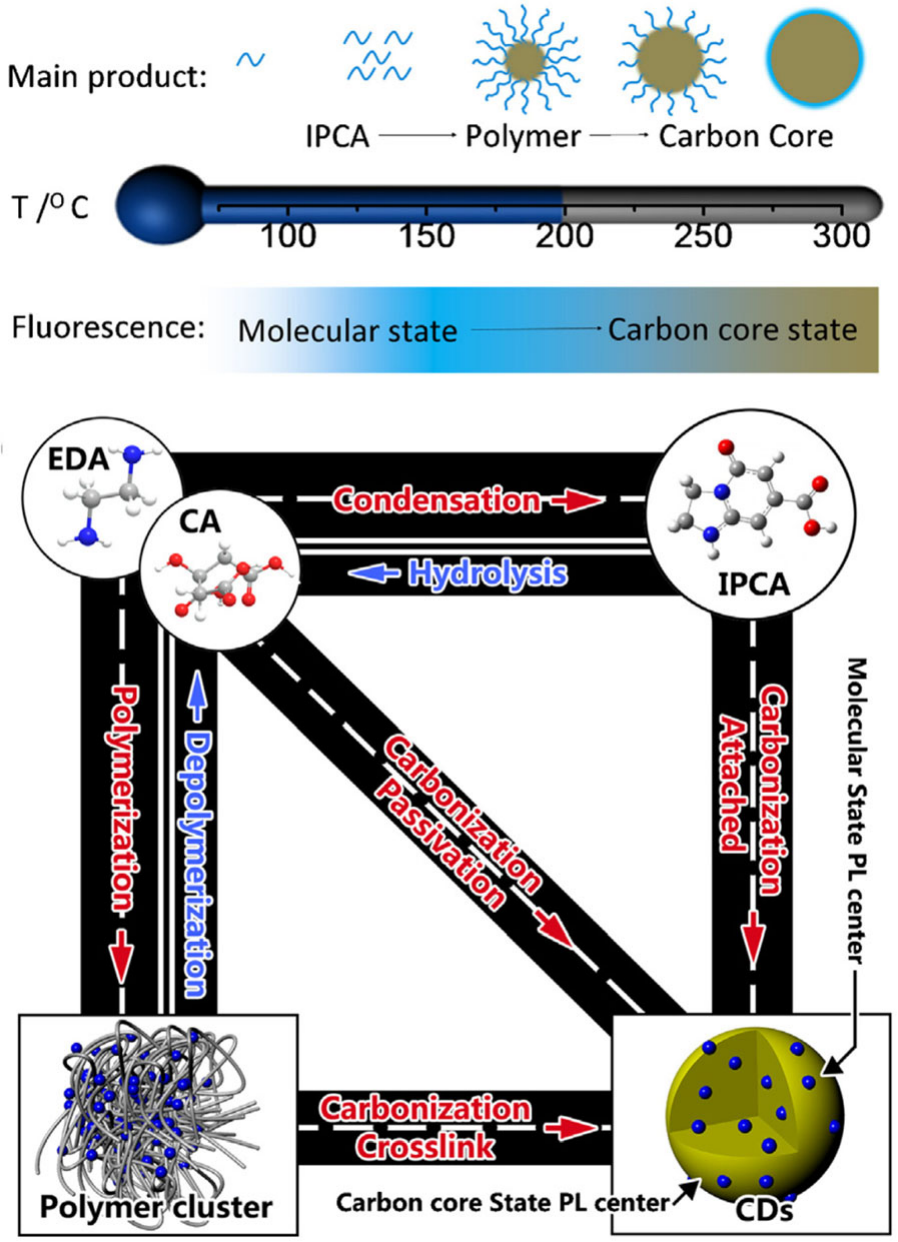
**a** Scheme showing bottom-up formation of CDs at different temperatures and **b** the relationship between different products [50]

Bottom-up methods synthesize CDs from various small carbon molecules including citrates, carbohydrates, and other green materials. In this method, it is easy to control the surface state, as well as the size, of the CDs [47–49]. The whole synthetic procedure is briefly described. The process is initiated from the carbonization of carbon precursors, which occurs concurrently with dehydration via heat-treatment through hydrothermal, microwave, or pyrolysis methods in concentrated acids (Fig. 3) [50–52]. The microwave-assisted hydrothermal method is common, and it is easy to synthesize CDs from various organic sources, including sucrose, glucose, saccharides, amino acids, and proteins, which can all be used as building blocks for CDs [53–56]. Because of the diversity of precursor materials, various functional groups remain after synthesis, and these are beneficial for enhancing the fluorescence of the CDs. It is also possible to create CDs by refluxing candle soot in a strong acid, where the oxidation by the acid is important for the dissolution of the soot [57]. However, the fluorescent products derived from small carbon molecules limit the mass production of CDs and lack quality control because of the heterogeneity.

## Physical Properties of Carbon Dots

### Structures

**Fig. 4:**
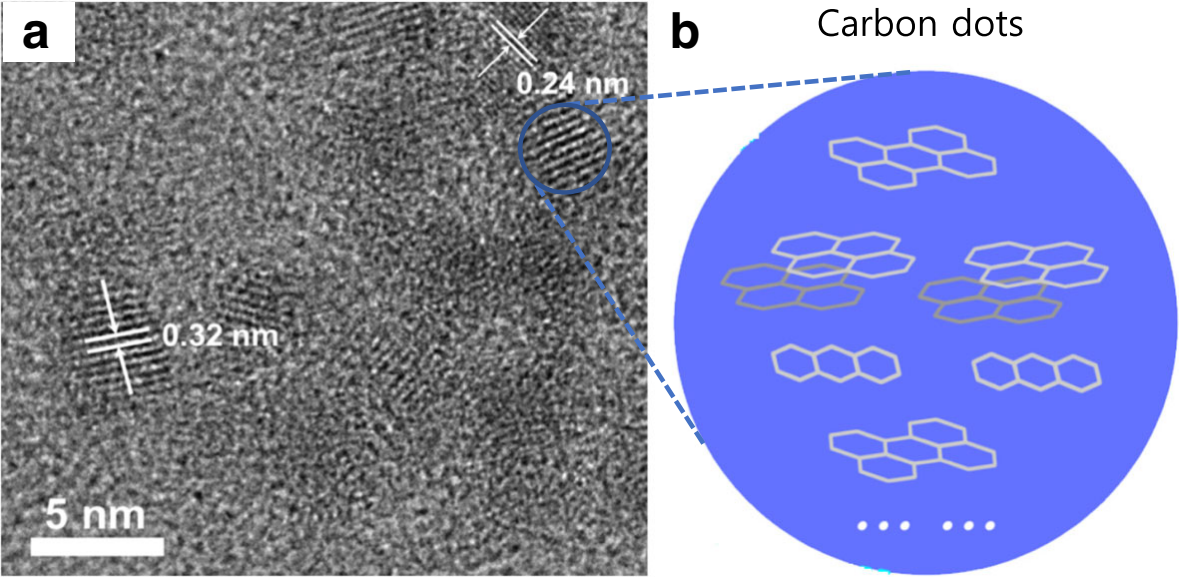
**a** High-resolution TEM images showing measurement of the space between lattices of CDs and **b** schematic representation of CDs with a carbogenic core containing sp^2^ carbon [33]

Understanding the structures of CDs is critical to understanding their key features, including fluorescence. CDs mostly have a graphitic in-plane lattice spacing of 0.18–0.24 nm and graphitic interlayer spaces of 0.32 nm (Fig. 4a). Although the detailed structure of the CDs varies depending on the raw materials and synthetic method, it is generally accepted that CDs are composed of carbon crystalline cores similar to sp^2^ carbon and amorphous clusters (Fig. 4b) [33, 58, 59]. Generally, the degree of crystallinity of CDs is lower than that of GQDs, and some CDs contain diamond-like sp^3^ carbon [37]. Raman spectroscopy reinforces these observations, and two peaks around 1350 and 1600 cm^−1^ are typically observed, indicating disordered sp^2^ carbon and crystalline graphitic carbon, respectively [33, 59]. In addition to the core carbon framework, different functional groups are usually introduced into CDs via surface passivation or functionalization, and these protect the surface and enhance the fluorescence of the CDs.

### Fluorescence

**Fig. 5:**
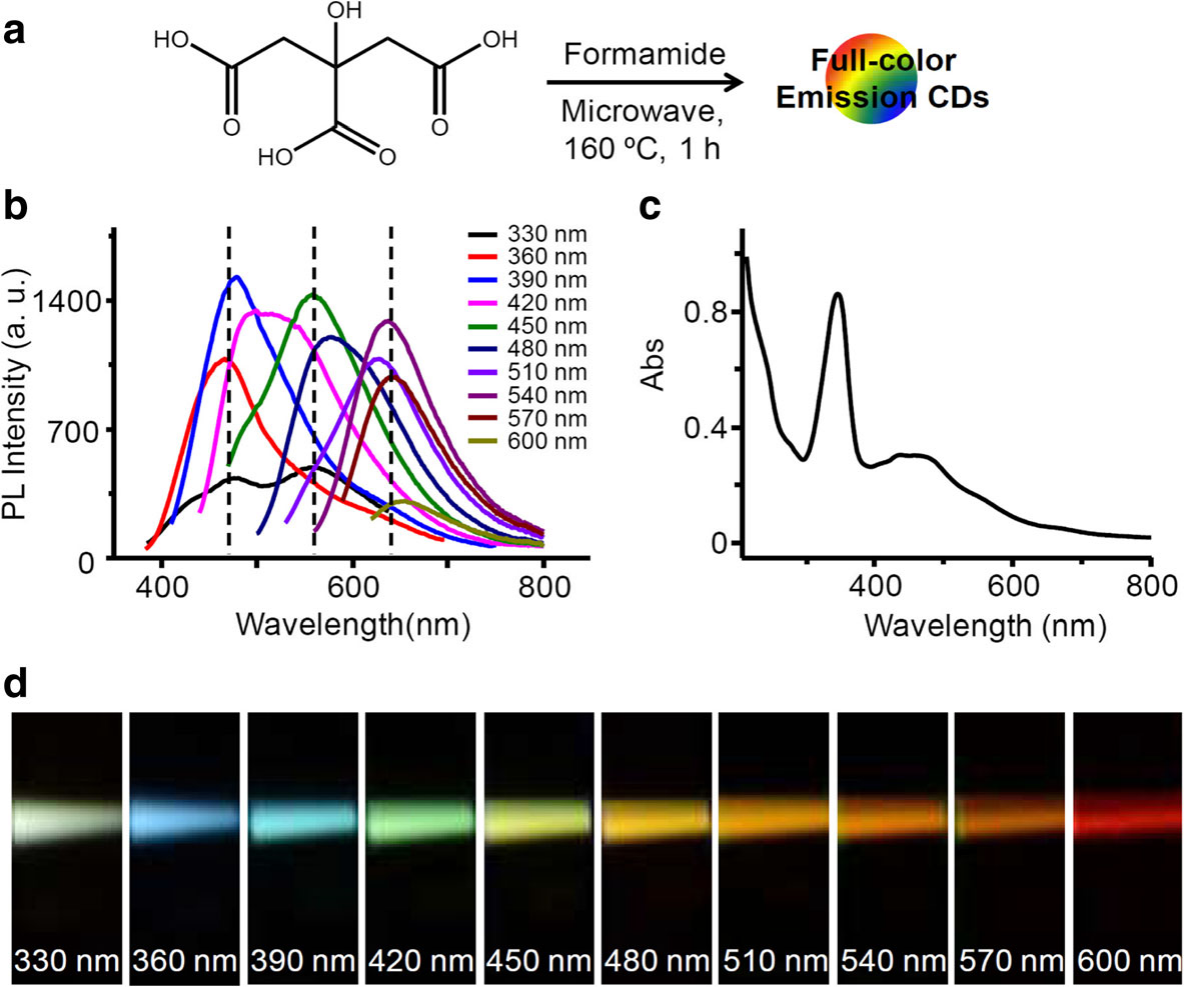
**a** Schematic of the preparation of the full-color-emission CDs. **b** Fluorescence spectra of the fluorescent CDs (F-CDs) under different excitation wavelengths. **c** UV-vis absorption spectra of the F-CDs. **d** Fluorescence emission photographs of the F-CDs recorded from 330 to 600 nm in 30 nm increments. All spectra and photographs were obtained in deionized H_2_O [60]

It is generally accepted that the surface state of CDs is closely correlated with their fluorescence. However, because of structural complexity of CDs, the exact underlying mechanism of CD fluorescence is unclear and requires clarification. Pan et al. addressed this question with full-color and blue-color CDs (Fig. 5) [60]. The optical properties of CDs, even those prepared from the same materials (mixtures with the same ratio of citric acids and formamide), can differ depending on the temperature and duration of the heat applied in the microwave hydrothermal method. That is, two different CD samples can display different fluorescence spectra. The CDs prepared at high temperatures for long reaction times showed a full color spectrum, whereas those created in a short period at low temperatures showed a blue color when irradiated with the same wavelength. This could be attributed to the differences in size of the CDs, which affects the emission profiles of CDs because, like semiconductor QDs, their emission depends on quantum confinement effects; i.e., as the size of QDs decreases, the energy gap between the valence shell and conduction band widens, and the emission wavelength decreases. However, the differences can also arise because of the surface state of the CDs, and investigation revealed that the full-colored CDs had more functional groups, including C=N/C=O and C-N groups, on their surfaces than the other sample [59, 60]. Consistent with previous study, the evidence suggests that the fluorescence of CDs is not caused by a single factor but arises from a combination of several factors such as size, surface passivation, functional groups, and heteroatoms [61].

### Surface Passivation and Doping

Pristine CDs, also called undoped CDs, have exposed carbon and oxygen sites after the initial synthesis step [33]. Passivation protects the carbon and oxygen-containing groups on the surface from interacting with other organic molecules, thus preserving the optoelectronic properties of the CDs. Polymeric PEG_1500N_ has been introduced onto CDs by acid treatment, and this has been shown to enhance the fluorescence of the CDs [32]. Surface passivation itself also contributes to the functionalization of CDs with no need for further modification. Many other materials have also been applied, such as different molecular weights of PEG, branched polyethyleneimine (b-PEI), and diamine-terminated oligomeric PEG, yielding polyamine-passivated CDs and CDs functionalized with free amines; this allows fluorescence tuning [62]. Different functional groups affect the energy levels of the CDs, which alter and enhance the light absorption and emissive spectrum of the probes. Additionally, surface modification also enables the modulation of the solubility of CDs in certain solvents. For example, the acid treatment of CDs generally results in the incorporation of carboxyl, carbonyl, and hydroxyl groups [32, 57].

**Fig. 6:**
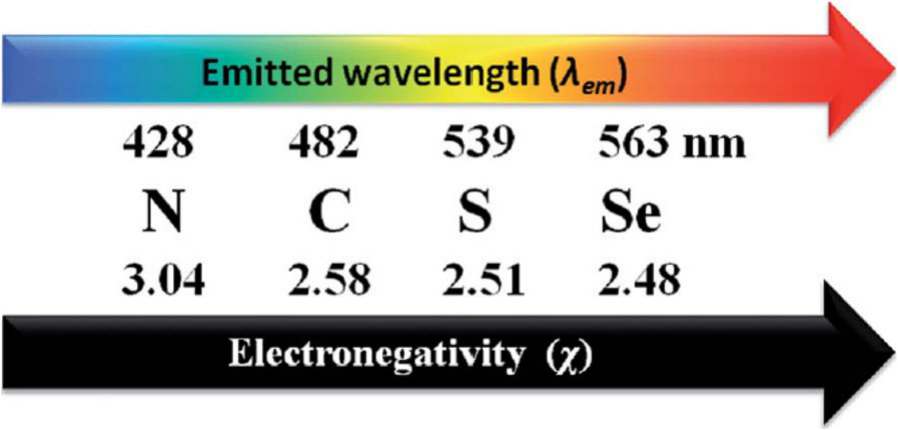
The relationship between the electronegativity of heteroatoms and the emission wavelength (λ_em_) of the doped CDs [64]

Burlinos et al. demonstrated the functionalization of CDs by one-step pyrolysis, in which a mixture of citric acid and different amines underwent thermal decomposition. In this system, citrate provided the carbon core, whereas the amines were attached as functional groups on the CDs [63]. Yang et al. reported a method for the large-scale preparation of heavy metal-doped CDs with tunable photoluminescence [64]. Initially, the carbon nanoparticles from Chinese ink were oxidized and cleaved simultaneously using an established process to obtain oxidized-CDs as precursors. Then, heteroatom (N, S, or Se)-doped CQDs were obtained by a one-step hydrothermal reduction and in situ doping treatment. The heavy metal-doped CQDs are just 1–6 nm size and have improved photoluminescence with different emission wavelengths depending on the electronegativity of the heteroatoms (Fig. 6). Moreover, these N- and S-doped CDs were very sensitive for the detection of Cu^2+^ and Hg^2+^, respectively [64].

**Fig. 7:**
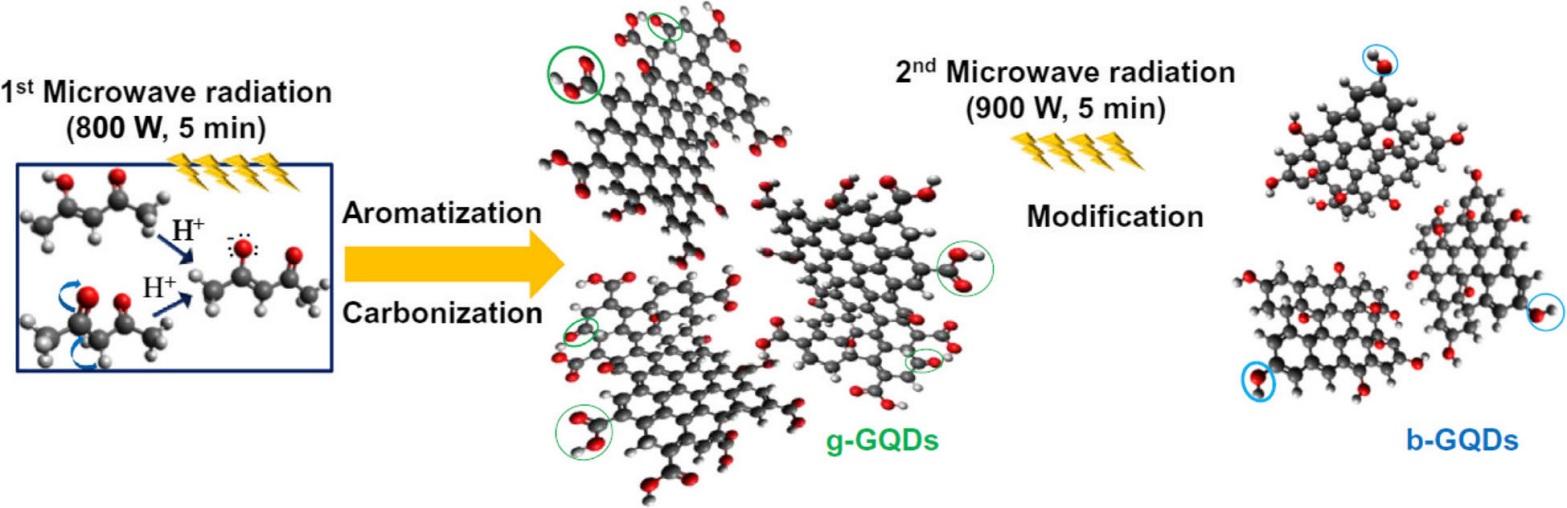
Schematic illustration of microwave bottom-up route for green-GQDs and blue-GQDs: green circles indicate carboxyl and carbonyl groups, and blue circles indicate hydroxyl groups [56]

Because graphene is a zero-band gap material, it is necessary to introduce optoelectronic properties to pristine graphene [65]. The incorporation of dopant atoms is a promising way to tune graphene’s fluorescence properties. Chemical functionalization also enables the band gap to be changed, and the changes in the band gaps result in shifts in the Fermi level [66]. Doping with more electronegative atoms than carbon, such as nitrogen, leads to a blue shift in emission, whereas doping with less electronegative elements than carbon, such as sulfur and selenium, shifts the fluorescence to red [64]. In particular, the introduction of oxygen, especially epoxy or hydroxyl groups, widens the bandgap of the sp^2^-hybridized carbon network. After CD formation, N-doped CDs were prepared by sequential treatment with organic carbon sources such as hydrazine, urea, hexamethylenetetramine, diethylamine, ethanolamine, and ethylenediamine, which increased the electron density, reduced the work function of the CDs, and resulted in a blue shift in emission. In addition, Umrao et al. reported a sequential bottom-up route to produce green and blue luminescent GQDs (g-GQDs and b-GQDs) by reversibly tailoring the size and functional groups via microwave carbonization and aromatization processes from acetylacetone as a starting organic solvent (Fig. 7) [56]. In contrast to initial green luminescent of g-GOD, the b-GQDs as the final product show only one emission peak at 433 nm and pH-independent blue luminescence because the two-step microwave irradiation process reduced the size and the oxygen-functional groups of the g-GQDs as an intermediate product.

## Decoration of CDs for the Detection of Heavy Metals

Heavy metals are often necessary and are rarely harmful to human health at low concentrations, but their accumulation can lead to a wide spectrum of debilitating diseases. In addition, heavy metal pollution, which is predominantly caused by Hg^2+^, As^3+^, Pb^2+,^ Cd^2+^, and Cu^2+^, is considered to be one of the most deleterious threats to the environment that could permanently undermine global sustainability [67]. Therefore, the development of versatile systems to monitor trace heavy metals continuously is crucial in modern society.

CDs are desirable candidates for use in potable detectors because of their abundance, high stability, low toxicity, and inexpensive nature [68–71]. Moreover, surface modification is facile and can be used to make the CDs soluble in water, as well as resulting in high fluorescence quantum yields, making them attractive candidates for biocompatible nanomaterials [72]. The binding and interaction between the probes and heavy metals causes changes in physicochemical properties of the fluorophores, including the fluorescence intensity, lifetime, and anisotropy, and provides a meaningful signal than can selectively indicate analytes with high sensitivity as a result of quantum confinement. Here, we outline recent studies related to different types of surface materials that will facilitate the application of CDs in heavy metal detection [73–77].

### Organic Molecules

The initially synthesized CDs exhibit no fluorescence and are poorly dispersed in polar solvents such as H_2_O and ethanol, which limits the utilization of fluorescent CDs as environmental probes or for biological applications for detecting heavy metals. Accordingly, numerous researchers have focused on the development of CDs to enhance their quantum yield and dispersibility in polar solvents. One easy way to achieve this is to incorporate various functional groups on the surface of the CDs. Zhu et al. reported a facile hydrothermal method using citric acid and ethylene diamine; interestingly, they investigated how changes in the ratio of the two precursors affected the quantum yield in response to Fe^3+^. They found that changing the ratio of the two components altered the number of incorporated hydroxyl and carboxyl residues. Thus, the final product showed different fluorescence intensities. Without amine groups, the quantum yield was less than 10%, and the maximum quantum yield was 60% in comparison to those of quinine sulfate. The fluorescence of the CDs was quenched in the presence of Fe^3+^, likely because of coordination between the hydroxyl groups of the CDs and Fe^3+^. The detection limit for Fe^3+^ was 1 ppm [78]. This result clearly suggests that the tuning of the functional groups is important for achieving optimal probe fluorescence. Sun et al. also reported the preparation of amine-functionalized GQDs from ammonia by hydrothermal treatment, and this increased the quantum yield by eight times compared to that of the native GQDs. In addition, the GQDs showed high selectivity to copper ions [79]. Dong et al. reported an effective method to detect trace amounts of Cu^2+^ ions using branched polyethyleneimine-functionalized CDs as fluorescent probes [80]. An increase in the fluorescence intensity occurred on exposure to Cu^2+^. Furthermore, they tested this probe in real river water samples, and it showed a linear response from a Cu^2+^ concentration of 0 to 9 μM; this sensor was affected by the pH, only showing sensitivity at pH 4.0, however.

One method to tailor carbon-based nanomaterials is the introduction of other atoms such as nitrogen and sulfur, thus changing the electronic properties. The doping of graphene with nitrogen forms N-graphene, which has different properties compared to pristine graphene. The nitrogen dopants affect the distribution of the charge and spin densities of the carbon atoms, thereby activating the graphene surface [81, 82]. Ju et al. reported that N-doped GQDs synthesized from citric acid and doped with hydrazine through a simple hydrothermal method that are sensitive to Fe^3+^, having a detection limit of 90 nM [83]. Thus, heteroatom doping can drastically change the electronic characteristics of GQDs, and the label-free sensitive and selective detection of Fe(III) ions could be performed in real water samples. Thus, this method provides a simple and low-cost route for the production of sensing platforms.

Nitrogen–sulfur co-doped CDs prepared from a single polymeric precursor as highly sensitive photoluminescent probes for mercury detection were developed by Mohapatra et al. The turn on–off fluorescence changed upon mercury addition, and this is attributed to the nonradiative electron transfer from the excited state to the d-orbital of the metal ion. The soft–soft and acid–base interactions between the sulfur part of the CD and Hg^2+^ make the fluorescent probe more specific and selective toward Hg^2+^, having a limit of detection of 0.05 nM for mercury ions [84]. In addition, Wang et al. reported the synthesis of boron-doped CDs (B-C-dots) by hydrothermal synthesis using ascorbic acid and boric acid as precursors. Due to the charge transfer between the chelate oxygen atoms on the CD surface, the strong fluoresce can be quenched by Cu (II) and Pb (II) ion [85].

**Fig. 8:**
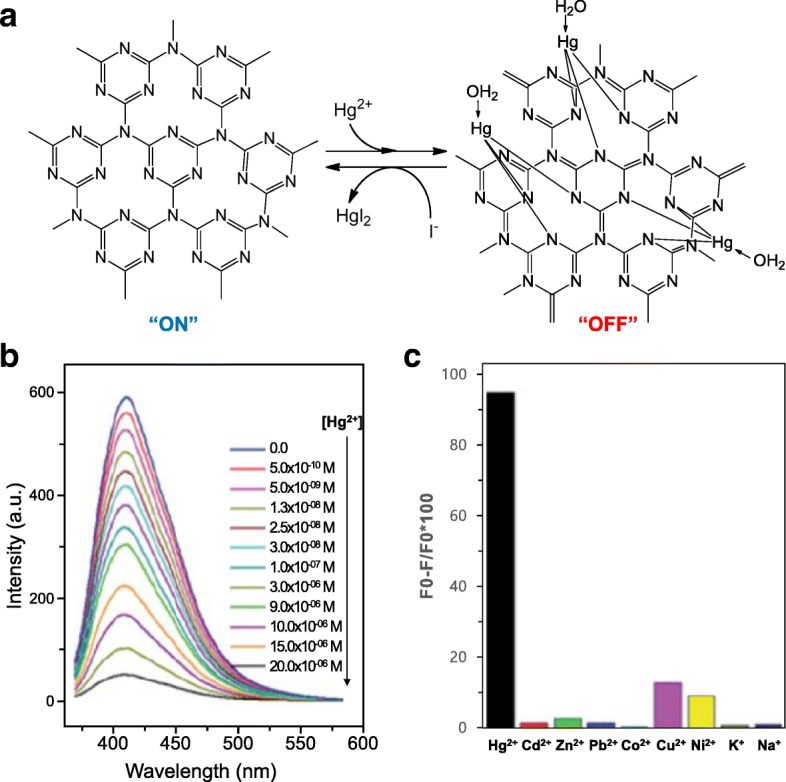
**a** Schematic of N-doped graphene-(Hg^2+^) complex and N-doped graphene on the addition of I-ions. **b** The change in the fluorescence emission of N-doped graphene (5 μg L^−1^) in water on the addition of Hg^2+^ ions. **c** Fluorescence quenching of Hg^2+^ ions compared to that of other metal ions [86]

Barman et al. reported highly blue fluorescent graphitic carbon nitride QDs (g-CNQDs) for the detection of mercuric and iodide ions. Mercury was chosen as a target because it causes a neurological syndrome called Minamata disease [86]. To synthesize the g-CNQDs, microwave-mediated synthesis was used with a formamide precursor. Because of their greater affinity toward nitrogen than carbon, their large radius, and their ability to form complexes with nitrogen, Hg^2+^ ions can affect the quenching of the fluorescence properties of g-CNQDs sensitively and selectively. The formation of the non-fluorescent cleating g-CNQD-(Hg^2+^)_*x*_ complex resulted in a non-fluorescent “OFF” state, whereas the addition of I^−^ ions changed this “OFF” state to an “ON” state, indicating that the formation of chelating Hg^2+^ complex had occurred (Fig. 8).

### Biomolecules and Natural Materials

**Fig. 9:**
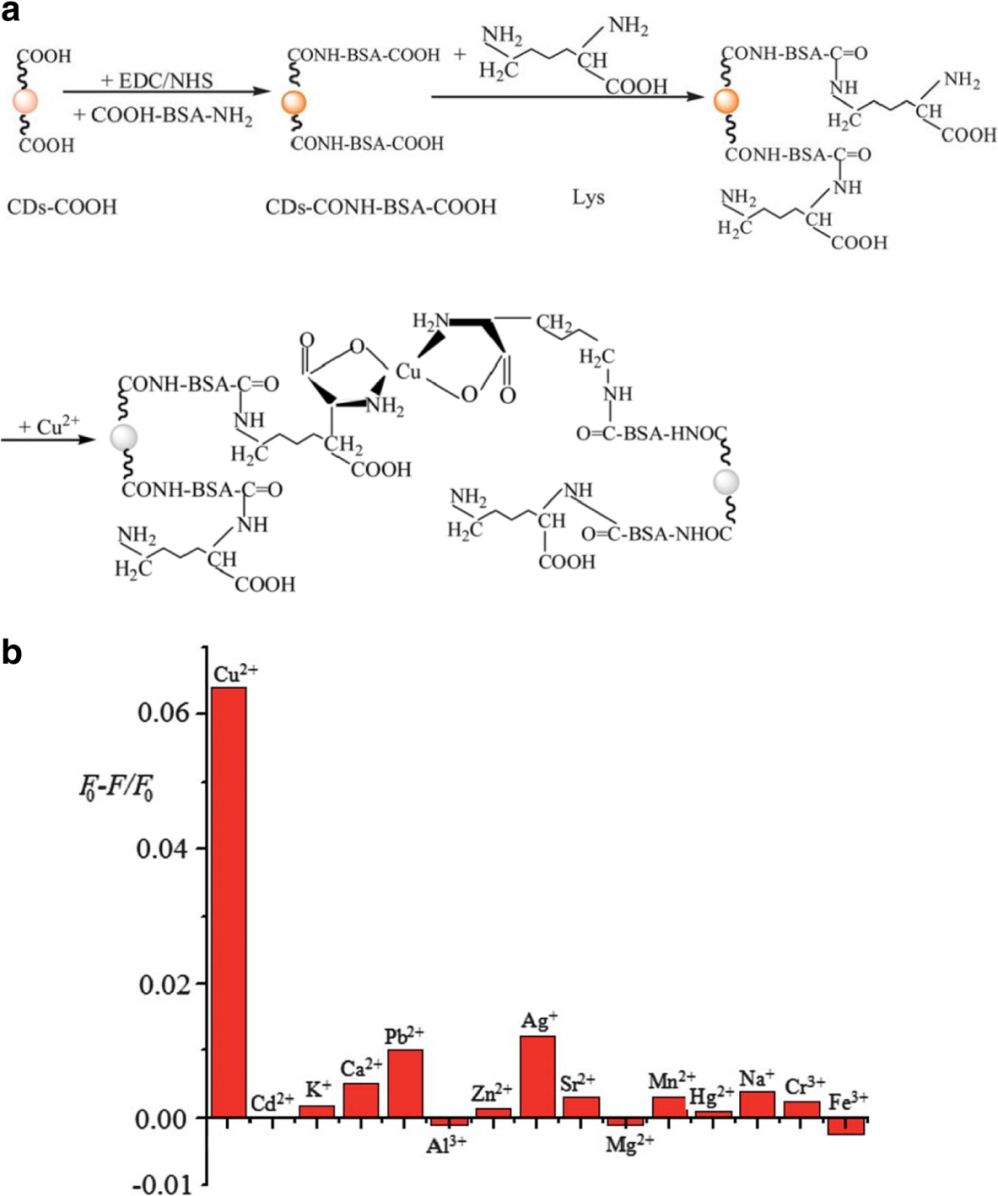
**a** Schematic of the CD modification with BSA and Lys and Cu^2+^ detection. **b** The selectivity of the CDs-BSA-lysine fluorescent probe toward 2 nmol Cu^2+^ in the presence of other cations under optimum conditions [87]

Biomolecules have great potential for the modification or synthesis of CDs when there are concerns regarding toxicity and biocompatibility. Various biochemical components produced in nature, including amino acids, oligosaccharides, and their macromolecules and derivatives, can be used. Liu et al. reported that lysine-coated CQDs modified with bovine serum albumin (CQDs-BSA-Lys) could be used for the detection of Cu^2+^ ions [87]. The synthesis of the pristine CDs was carried out using a mixture of glucose and PEG_200_ by microwave treatment. BSA was mixed with a coupling reagent with gentle stirring, resulting in carbodiimide formation. The subsequent addition of lysine greatly enhanced the fluorescence of the CQDs-BSA, probably because of the interactions between the carboxylic acids and amines from both BSA and lysine, as well as the formation of a coating layer, which likely reduced the surface defects on the CDs. The CDs were tested for their function as a copper-selective probe in the presence of various heavy metals, and the probe showed specificity for copper, detecting Cu^2+^ concentrations of 2 nmol (Fig. 9). The Cu^2+^ ions appear to form multiple coordination complexes around the carboxylic acids and amines of lysine in the CQDs and glycine on the partially uncoated CQDs [87].

Valine-functionalized GQDs (Val-GQDs) were synthesized by simultaneous mixing with citric acid via thermal pyrolysis [88]. The base GQDs were formed from pyrolyzed citric acid through dehydration and carbonization, and the incorporated valine led to changes in the fluorescence. The quantum yield of the Val-GQDs was increased fourfold compared to that of pristine GQDs. The increase in the quantum yield was caused by changes in the steric and electronic properties, likely induced by the increase of nitrogen moieties in pyridine and pyrrole groups formed after the functionalization with valine [88, 89]. Interestingly, the presence of valine moieties in the Val-GQDs resulted in a more sensitive fluorescent response to Hg^2+^, showing a detection limit of 0.4 nM (signal-to-noise ratio = 3) and a sensitivity 14-times greater that of the unmodified GQDs.

**Fig. 10:**
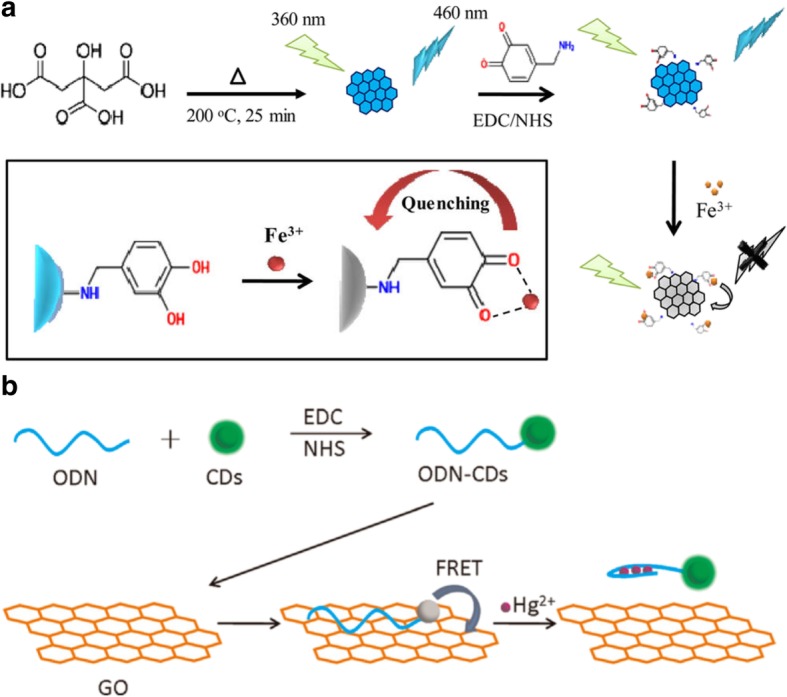
Schematic of the **a** preparation of a dopamine-functionalized GQD (DA-GQD) sensor [89], and **b** the proposed mechanism for Fe^3+^ ions and the FRET-based sensor system for Hg^2+^ detection using CDs and GO [91]

Chowdhury et al. selected dopamine, a well-known neurotransmitter derived from amino acids, as a conjugator [90]. Their idea was based on the fact that dopamine forms Fe^3+^ complexes in the body, which would enhance the fluorescence and the sensitivity to Fe^3+^ of GQDs. The GQDs were fabricated by the pyrolysis of citric acid, followed by covalent conjugation with dopamine. After the addition of ferric ions, complexes with the catechol moiety of dopamine formed, followed by oxidation to o-semiquinone, resulting in a decrease in the fluorescence intensity of the GQDs (Fig. 10a). The fluorescence intensity changed linearly within a range of 0–1.5 μM, and the lowest limit of the detection was 7.6 nM. Cui et al. [91] prepared and tested a fluorescence resonance energy transfer (FRET)-based system to detect Hg^2+^ using oligodeoxyribonucleotide-conjugated CDs (ODN-CDs). The thymine-rich 22-base-pair nucleotides on the CDs act as electron donor and the GO acts as an electron acceptor. In the absence of Hg^2+^, the energy of the fluorescence emitted from the oligomers on the CDs was absorbed into GO, and its fluorescence was quenched. On the other hand, in the presence of Hg^2+^ ions, the thymine in the oligomers selectively interact with Hg^2+^, forming self-hybridized oligomers. The folded structure of the ODN-CDs prevents the interaction with GO, so the quenched fluorescence is recovered (Fig. 10b) [91]. Therefore, the fluorescence was recovered as the mercury concentration increase, and this system could monitor the Hg^2+^ concentration in a linear range from 5 to 200 nM with selectivity for mercury over other cationic metals except Fe^2+^.

Chitosan is a natural material and is the main component of the outer shells of shellfish such as crabs. Its abundance and biosafety are advantageous for its use as a CD precursor, and studies have shown that it can be used to produce N-doped CDs in a simple process because it provides both carbon and nitrogen together [91]. This method overcomes the general problems suffered by CDs derived from natural materials, which often have low quantum yields, and the CDs showed a 31.8% quantum yield. In addition to smartphone applications, these materials also have possible applications as portable detection probes for Hg^2+^, having a detection limit of 80 nM. The N-doped CDs showed strong fluorescence near 440 nm without Hg^2+^, whereas the fluorescence was greatly quenched in the presence of Hg^2+^. Its fluorescence decay was linear within a range of 80–300 μM Hg^2+^ [92].

Sahu et al. reported a green synthesis for the fabrication of highly fluorescent CDs from natural source, the leaves of *Ocimum sanctum*, in a single step. The eco-friendly prepared CDs have excellent selectivity toward Pb^2+^ ions with a detection limit of 0.59 nM and linear detection range of 0.01–1.0 μM and good cell-permeability and low cytotoxicity, thus effectively used for the fluorescence cell imaging [93].

### Metal Nanoparticles

Novel metal nanoparticles, such as those of Au, Ag, and Pt, exhibit distinctive surface plasmon resonance (SPR) peaks depending on their size and shape. Interestingly, composites of carbon-based nanomaterials and novel metal nanoparticles have been studied because of their characteristic optical properties. Noble metal clusters can be immobilized with great stability through hybridization between the sp^2^ dangling bonds at the defect sites of graphene sheets and the clusters. After immobilization, the fluorescence of the GQDs can be quenched by these metal nanoparticles or clusters of ions can form by charge transfer processes [94]. Inspired by these phenomena, Ran et al. synthesized Ag nanoparticles decorated with GQDs for the rapid, and sensitive detection of Ag^+^ and bithiols [95]. The formation of AgNPs on GQDs quenches the fluorescence of the GQDs, and the addition of bithiols causes a further turn-off phenomenon via their strong interactions through the formation of Ag–S bonds.

**Fig. 11:**
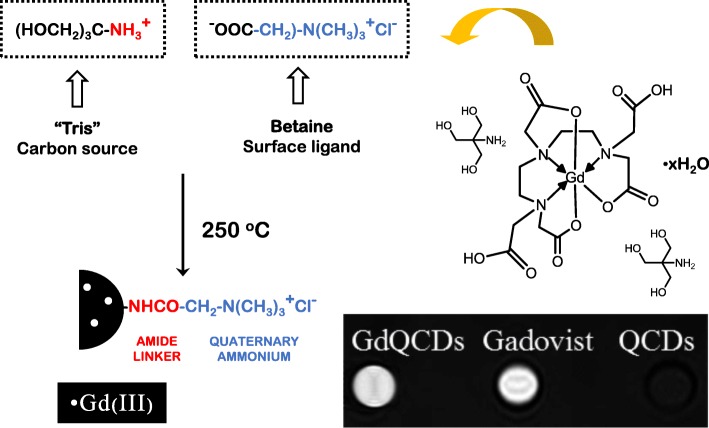
Synthesis of Gd-QCDs. The Gd(III) centers are immobilized in the carbonaceous matrix through coordination by residual O and N heteroatoms [97]. The inset shows the MRI positive contrast effects in T1-weighted images of the Gd-QCDs and the commercial Gd-based contrast agent-Gadovist

Ting et al. reported novel conjugates of cysteamine-capped gold nanoparticles (AuNPs) and GQDs, and these were used for the sensitive electrochemical detection of Hg^2+^ and Cu^2+^ with detection limits of 0.02 and 0.05 nM, respectively [96]. The Hg^2+^ ions are pre-concentrated onto the electrode by applying a negative voltage (− 0.2 V and 120 s), and the negatively charged hydroxyl and carboxyl groups interact with Hg^2+^ because of the formation of R-COO-(Hg^2+^)-OOC-R groups, as well as the initial binding of mercury onto AuNPs. In the case of Cu^2+^ ions, the anodic stripping voltage of copper occurs at 0 V, meaning that it is clearly separated from that of mercury ions and implying the possibility of the simultaneous detection of the two-ion species. In addition, Bourlinos et al. presented the synthesis of ultrafine sized Gd(III)-doped CDs with dual fluorescence/magnetic resonance imaging (MRI) character via the thermal decomposition of a precursor composed of an organic salt and a gadolinium(III) complex. The dots are water-dispersible, display bright fluorescence in the visible range upon light excitation, and show strong T1-weighted MRI contrast comparable to that of commercial Gadovist, as well as possess low cytotoxicity (Fig. 11) [97].

Zhang et al. reported an efficient CQD-gold nanocluster (CQDs/AuNCs) nanohybrid prepared by a one-step hydrothermal treatment with alanine and histidine. The hybrid materials were used for ratiometric fluorescent probe for sensitive and selective sensing of CD (II) ions with a detection limit of 32.5 nM. Interestingly, the quenched fluorescence by Cd^2+^ can be gradually recovered upon the concentration of l-ascorbic acid (AA)with a detection limit of 105 nM and this fluorescent “on-off-on” system can be practically used for the excellent detection to Cd^2+^ and AA in lake water and in human serum, respectively [98].

## Conclusion

Much research into carbon-based quantum dots has been reported in the last few decades, and a wide range of synthetic methods and characterization techniques have been used. In most cases, studies of these fluorescent materials have focused on their bioimaging applications. Although some heavy metals are essential in the human body, excess heavy metals cause disease, for example, Minamata disease and Itai-itai disease. Thus, recent progress in fluorescent CDs has opened the possibility of developing portable detectors for dangerous heavy metals, and we have outlined recent studies related to surface materials that will enable the development of heavy metal sensors as a portable device [99]. Moreover, the progress in biocompatible fluorescent CDs enables harmless onsite detection as well as the color-mediated analysis provides easy interpretable readout even for non-professional persons. However, relatively low solubility of CDs in water remains challenges and low cost for fabricating devices is another requirement for the use of CDs in various fields, even though many synthetic methods have been developed. In addition, the exact mechanism for different photoluminescent which depends on the synthetic method and raw carbon sources should be more cleared. We hope that this review will inform researchers about the recent progress in carbon-based quantum dots for heavy metal sensing, leading to develop new eco-friend and cost-effective synthetic methods and practical use.

## Data Availability

It is a review article that gives a comprehensive study about the recent progress in carbon-based quantum dots for fabrication, features, and application in heavy metal sensing.

## Abbreviations

AuNPs: Gold nanoparticles
B-C-dots: Boron-doped CDs
CD: Carbon dots
CQDs: Carbon quantum dots
CQDs/AuNCs: CQD-gold nanocluster
CQDs-BSA-Lys: Lysine-coated CQDs modified with bovine serum albumin
g-CNQDs: Graphitic carbon nitride QDs
g-GQDs and b-GQDs: Green and blue luminescent GQDs
GQDs: Graphene quantum dots
MRI: Magnetic resonance imaging
MWCNTs: Multiwall carbon nanotubes
ODN-CDs: Oligodeoxyribonucleotide-conjugated CDs
PEG1500N: Diaminopolyethylene glycol
PPEI-EI: Poly(propionylethyleneimine-co-ethyleneimine)
QDs: Quantum dots
SPR: Surface plasmon resonance
SWNTs: Single-walled nanotubes
TEM: Transmission electron microscopy
Val-GQDs: Valine functionalized GQDs
